# Identification and functional analysis of lactic acid metabolism-related differentially expressed genes in hepatocellular carcinoma

**DOI:** 10.3389/fgene.2024.1390882

**Published:** 2024-04-16

**Authors:** Haiyan Li, Fuchu Qian, Shengjie Bao

**Affiliations:** ^1^ Department of Laboratory Medicine, Huzhou Maternity and Child HealthCare Hospital, Huzhou, Zhejiang, China; ^2^ Department of Precision Medicine, Affiliated Central Hospital Huzhou University, Huzhou Central Hospital, Huzhou, Zhejiang, China; ^3^ Huzhou Key Laboratory of Precision Medicine Research and Translation for Infectious Diseases, Huzhou Central Hospital, Huzhou, Zhejiang, China; ^4^ Department of Laboratory Medicine, The First Affiliated Hospital of Huzhou University, Huzhou, Zhejiang, China

**Keywords:** lactic acid metabolism, gene, hepatocellular carcinoma, prognosis, bioinformatics

## Abstract

**Background:** Hepatocellular carcinoma (HCC) is a malignant tumor with high morbidity and mortality rate that seriously threatens human health. We aimed to investigate the expression, prognostic value, and immune cell infiltration of lactic acid metabolism-related genes (LAMRGs) in HCC using bioinformatics.

**Methods:** The HCC database (The Cancer Genome Atlas–Liver Hepatocellular Carcinoma) was downloaded from the Cancer Genome Atlas (TCGA). Differentially expressed genes (DEGs) between normal and tumor groups were identified. The LAMRGs were obtained from literature and GeneCards and MSigDB databases. Lactic acid metabolism-related differentially expressed genes (LAMRDEGs) in HCC were screened from the DEGs and LAMRGs. Functional enrichment analyses of the screened LAMRDEGs were further conducted using Gene Ontology (GO) analysis, Kyoto Encyclopedia of Genes and Genomes (KEGG) analysis, and Gene Set Enrichment Analysis (GSEA). The genes were used in multivariate Cox regression and least absolute shrinkage and selection operator (LASSO) analyses to construct a prognostic model. Then, a protein-protein interaction network was constructed using STRING and CTD databases. Furthermore, the CIBERSORTx online database was used to assess the relationship between immune cell infiltration and hub genes.

**Results:** Twenty-eight lactic acid metabolism-related differentially expressed genes (LAMRDEGs) were identified. The GO and KEGG analyses showed that the LAMRDEGs were related to the prognosis of HCC. The GSEA indicated that the LAMRDEGs were significantly enriched in tumor related pathways. In the multivariate Cox regression analysis, 14 key genes (E2F1, SERPINE1, GYS2, SPP1, PCK1, CCNB1, CYP2C9, IGFBP3, KDM8, RCAN1, ALPL, FBP1, NQO1, and LCAT) were found to be independent prognostic factors of HCC. Finally, the LASSO and Cox regression analyses showed that six key genes (SERPINE1, SPP1, CCNB1, CYP2C9, NQO1, and LCAT) were associated with HCC prognosis. Moreover, the correlation analyses revealed that the expression of the six key genes were associated with immune infiltrates of HCC.

**Conclusion:** The LAMRDEGs can predict the prognosis and may be associated with immune cells infiltration in patients with HCC. These genes might be the promising biomarkers for the prognosis and treatment of HCC.

## Introduction

Liver cancer is the sixth most prevalent cancer and the third leading cause (8.3%) of cancer-related deaths worldwide (Arnold et al., 2020). Hepatocellular carcinoma (HCC) is the most common liver cancer with a high mortality rate (Liver, 2018; Ding et al., 2019). Although progress in the diagnosis and treatment of HCC has improved the survival rate of patients with HCC, poor prognosis remains a challenge because most patients are diagnosed with HCC at an advanced stage due to a lack of early diagnostic and robust prognostic biomarkers (Yang et al., 2019). Therefore, there is an urgent need to identify new prognostic biomarkers to prolong the survival time of patients with HCC and explore the underlying molecular mechanisms of HCC to develop novel therapeutic targets.

Metabolic alterations are closely related to the occurrence, development, and high invasiveness of tumors (Somarribas Patterson and Vardhana, 2021). Increased lactic acid levels in the tumor microenvironment (TME) play a vital role in oncogenesis. Lactic acids not only a byproduct of glycolysis but also a critical modulator of normal and malignant cell signaling pathways (Vinasco et al., 2019; Brown et al., 2020). In an anaerobic environment, the production of high levels of lactic acid by tumor cells serves as a signal that promotes cancer cell proliferation, invasion, metastasis, and immune evasion and contributes to carcinogenesis (Brown and Ganapathy, 2020; Bergers and Fendt, 2021). Furthermore, lactic acid can establish metabolic coupling between cancer and adjacent cells to maintain tumor growth, andincrease the malignant phenotypes of tumors (Locasale, 2012). Tumor cells may metabolize lactic acid or transfer it to surrounding cancer, immune, and vascular endothelial cells, affecting the biological characteristics of the surrounding cells and resulting in metabolic reconfiguration (Hayes et al., 2021).

In recent years, the inhibition of lactic acid metabolism has proven to be a potential therapeutic approach for cancer (Doherty and Cleveland, 2013; Zeng et al., 2020). Previous studies have shown that lactic acid metabolism-related genes (LAMRGs) and lncRNAs play critical role in HCC (Li et al., 2021; Guan et al., 2022). However, the clinical significance and underlying mechanisms of lactic acid metabolism related differentially expressed genes (LAMRDEGs) in HCC remain poorly understood.

In this study, we aimed to screen LAMRDEGs in The Cancer Genome Atlas–Liver HCC (TCGA-LIHC) cohort from TCGA database and validated these genes using datasets from the Gene Expression Omnibus (GEO). We also investigated the functional enrichment of LAMRDEGs and visualized their signaling pathways. Furthermore, Cox regression and least absolute shrinkage and selection operator (LASSO) regression analyses were performed to explore potential prognostic biomarkers of HCC. Moreover, correlation between the LAMRDEGs and immune-cell infiltration, and chemotherapy drug sensitivity were further investigated. The current findings may provide novel insights into improve diagnosis, treatment, prognosis for HCC.

## Materials and methods

### Dataset download and candidate differentially expressed genes (DEGs) screening

Data regarding TCGA-LIHC cohort from TCGA (https://portal.gdc.cancer.gov/) was downloaded using the TCGAbiolinks package (Colaprico et al., 2016). The RNA-seq data (normal: 50, tumor: 374) of HCC patients were obtained. The expression profiles were standardized in fragments per kilobase per million (FPKM) format and log2-transformed, while corresponding clinical and survival information of the HCC patients were collected from the UCSC Xena browser (http://genome.ucsc.edu) (Goldman et al., 2020). In this study, the TCGA-LIHC dataset was severed as the training set.

Additionally, we downloaded HCC related microarray data of GSE25097 (Ivanovska et al., 2011) (https://www.ncbi.nlm.nih.gov/geo/query/acc.cgi?acc=GSE25097) and GSE54236 (Zubiete-Franco et al., 2019) (https://www.ncbi.nlm.nih.gov/geo/query/acc.cgi?acc=GSE54236) datasets from the GEO (https://www.ncbi.nlm.nih.gov/geo/) database using the GEOquery (Davis and Meltzer, 2007) package. The microarray platform for the GSE25097 dataset was GPL10687. This datasets included transcriptome profiles of 268 HCC tumor tissue samples. The microarray platform for the GSE54236 dataset was GPL6480. This datasets included gene expression of 81 HCC tumor tissue samples. The GSE25097 and GSE54236 datasets were served as the verification datasets for analysis.

The LAMRGs were collected from the GeneCards (https://www.genecards.org/) (Stelzer et al., 2016) and MSigDB database (https://www.genecards.org/) (Liberzon et al., 2015). We used the keyword “lactic acid metabolism” as the search keyword to obtain 1,986 LAMRGs from the GeneCards database. The same keyword was used to obtain 194 genes LAMRGs from the MSigDB database. In addition, 22 LAMRGs were obtained from literature (Zhao et al., 2022). Finally, we intersected the LAMRGs from the three sources to obtain 2139 genes related to lactic acid metabolism (Supplementary Table S1).

The limma package was used to screen the DEGs from expression profile of TCGA-LIHC dataset. The threshold was set as | logFC | > 2 and p. adjust <0.05. To obtain the LAMRDEGs, all DEGs from TCGA-LIHC datasets and LAMRGs were intersected and displayed using a Venndiagram. Volcano plots of the differences were generated using the “ggplot2”package. The heatmap regarding the LAMRDEGs was drawn using R package “pheatmap.”

### Function and pathway enrichment for the DEGs

The R package “clusterProfiler” (Yu et al., 2012) was used to conduct Gene Ontology (GO) analysis (Mi et al., 2019) and Kyoto Encyclopedia of Genes and Genomes (KEGG) (Kanehisa and Goto, 2000) enrichment analysis regarding the LAMRDEGs. The selection criteria were set as *p*. adjust< 0.05 and false discovery rate (FDR) < 0.25. The Benjamini-Hochberg method was used for *p* correction.

### Gene set enrichment analysis (GSEA) for the DEGs

The “c2. cp.all.v2022.1. Hs.symbols.gmt” gene sets were obtained from the MSigDB database (Liberzon et al., 2015). The “clusterprofiler” package was used for the GSEA (Subramanian et al., 2005) analysis of the DEGs obtained from TCGA-LIHC dataset. Furthermore, the significant enrichment standards were *p*. adjust< 0.05 and FDR <0.25.

### Construction of the cox regression model for predicting prognosis

We identified 14 key prognostic-related genes from the LAMRDEGs using Cox regression model. The expression of the 14 key genes was then included in the univariate Cox regression model and a forestplot was drawn. These 14 key genes were included in the multivariate Cox regression model, and a nomogram model was established to predict the 1-year survival of patients with HCC. Finally, a calibration curve was constructed to evaluate the prediction ability of the model. The “rms” package was used to construct the nomogram and calibration curves. We used the R package ggDCA (Tataranni and Piccoli, 2019) to conduct decision curve analysis (DCA) to evaluate the effect of the nomogram model in predicting 1-, 3-, and 5-year survival outcomes in patients with HCC. The formula for calculating the prognostic risk scores was:

$$\text{risk}Score=\sum_{i}Coefficient\left({gene}_{i}\right)*mRNAExpression\left({gene}_{i}\right)$$

### Gene Set Variation Analysis (GSVA) Based on the Cox Model

We obtained the gene set “H.A.v7.4. Symbols.gmt” from the MSigDB database (Liberzon et al., 2015), and conducted GSVA (Hänzelmann et al., 2013) regardingthe HCC samples in TCGA-LIHC dataset. We divided the HCC samples into high- and low-risk groups using a Cox model. The functional differences in the enrichment pathways between the high- and low-risk groups were analyzed.

### Construction of the LASSO regression model

To obtain the model of key genes in TCGA-LIHC dataset, we used the glmnet package (Yang et al., 2022) based on 14 key genes to conduct a LASSO regression (Cai and van der Laan, 2020). Through the LASSO model, we screened six key genes, visualized the results of the LASSO regression and presented the expression levels of each gene between the two groups in the diagnostic model.

### Protein-protein interactions networks analysis

The STRING database (Szklarczyk et al., 2015) was used to construct a protein-protein interaction (PPI) network using hub genes. In addition, we used the Comparative Toxicogenomics Database (CTD) (Davis et al., 2021) (http://ctdbase.org/) to predict potential drugs or small molecule compounds that could interact with hub genes. Cytoscape software was used to visualize the mRNA-drug interaction network.

### Analysis of immune cell infiltration

We excluded samples with no prognostic information and obtained expression profile data from TCGA-LIHC dataset. The information on the high- and low-risk groups in TCGA-LIHC dataset was obtained using a risk score model. The gene expression matrix of the dataset was uploaded to CIBERSORTx (https://cibersortx.stanford.edu) (Steen et al., 2020). Combined with the immune cell marker matrix, an immune cell infiltrating matrix was obtained (filter standard, *p* < 0.05). We then filtered out the data with an enrichment score of immune cells >0 and obtained the immune cell infiltration matrix. Finally, we acquired immune cells with significant differences in infiltration between the high- and low-risk groups using the LASSO model and plotted a heatmap and accumulation histogram of the correlation between the six key genes and these immune cells.

### Statistics analysis

All data processing and analyses were performed using the R software (version 4.1.2). To compare two groups of continuous variables, the independent Student’s t test was used to estimate the statistical significance of normally distributed variables. The Mann-Whitney U test (Wilcoxon rank test) was used to analyze the differences between non-normally distributed variables. The Kruskal-Wallis test was used to compare three or more groups. Furthermore, the Chi-square test or Fisher’s exact test was used to compare the differences between categorical variables. Correlations between the different genes were assessed using Spearman’s correlation analysis. Kaplan-Meier (K-M) analysis was used to evaluate the survival value of the key genes. The level of statistical significance was set at a *p*-value <0.05.

## Results

### Identification of the LAMRDEGs

**FIGURE 1:**
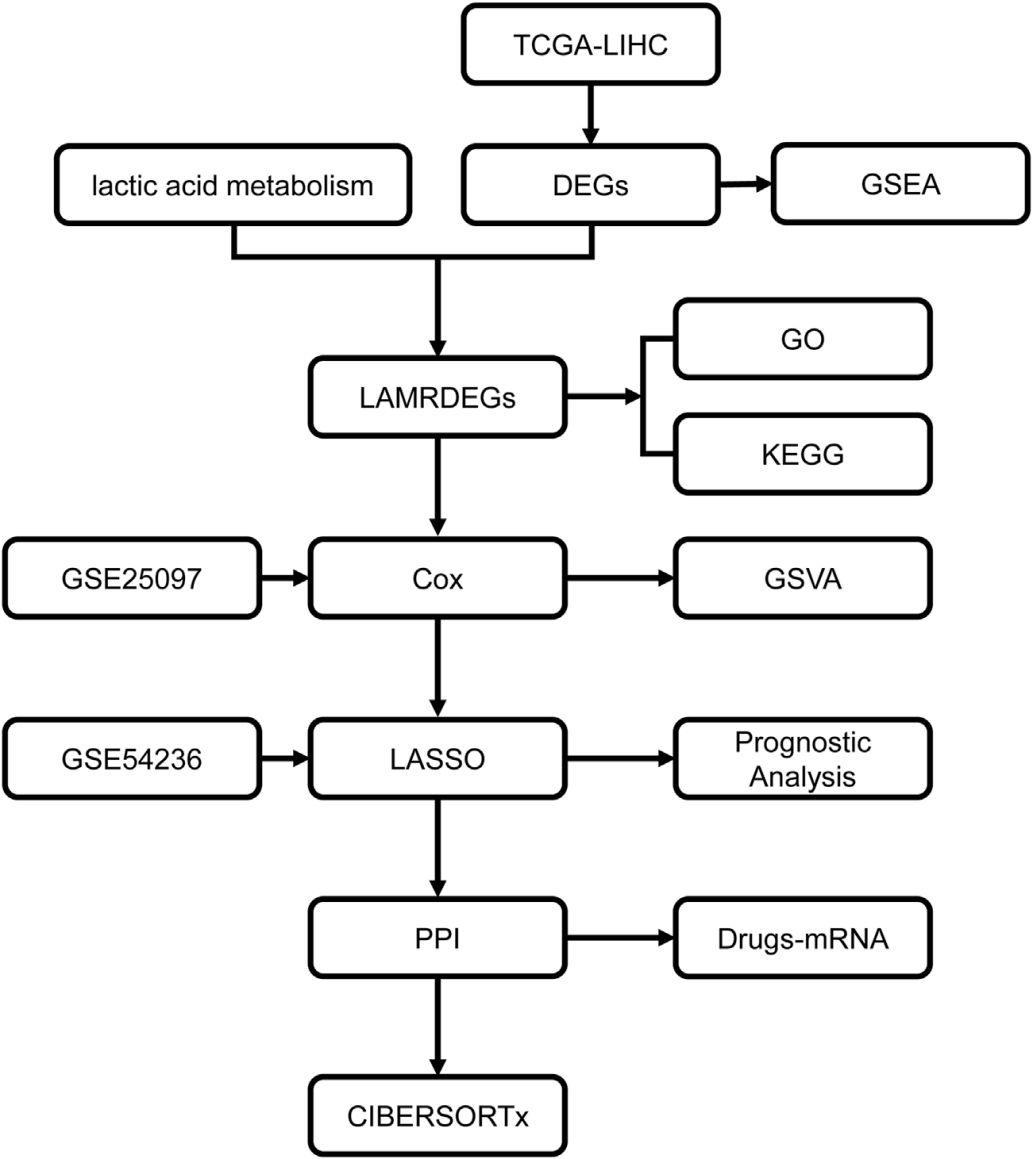
Workflow chart regarding the analyses in this study TCGA, the Cancer Genome Atlas; LIHC, liver hepatocellular carcinoma; DEGs, differentially expressed genes; LAMRDEGs, lactic acid metabolism-related differentially expressed genes; PPI, protein-protein interaction; GO, Gene Ontology; KEGG, Kyoto Encyclopedia of Genes and Genomes; GSEA, Gene Set Enrichment Analysis; GSVA, Gene Set Variation Analysis.

**FIGURE 2:**
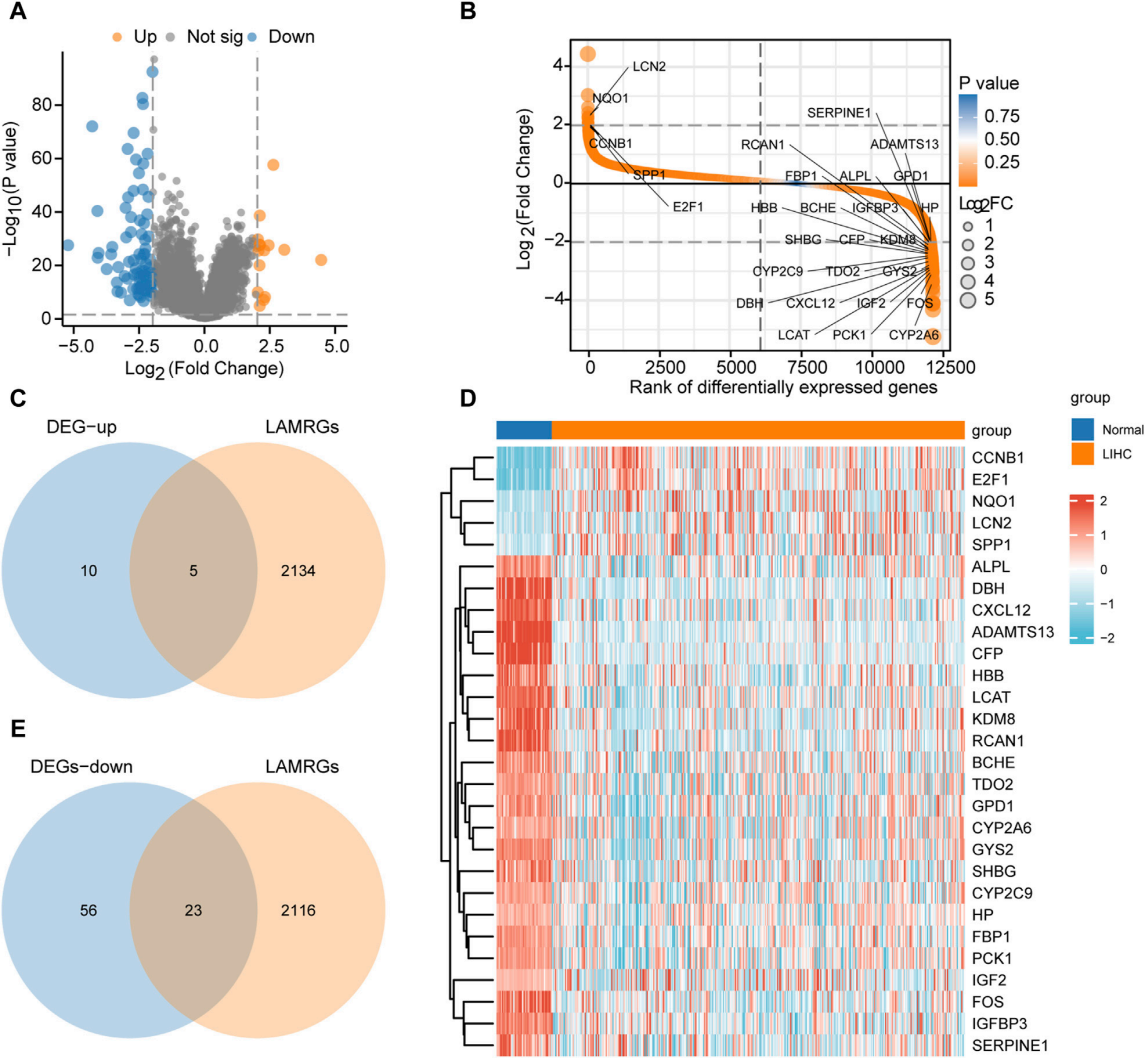
Differential gene analysis of TCGA-LIHC dataset **(A)**. Differential gene volcano plot of the LIHC and normal groups in TCGA-LIHC dataset. **(B)**. Rank of differential genes of the LIHC and normal groups in TCGA-LIHC dataset. **(C)**. Venn diagram regarding the upregulated DEGs in TCGA-LIHC dataset and LAMRGs. **(D)**. Heatmap regarding the LAMRDEGs between the LIHC and normal groups in TCGA-LIHC dataset. **(E)**. Venn diagram regarding the downregulated DEGs in TCGA-LIHC dataset and LAMRGs. LAMRDEGs, lactic acid metabolism-related differentially expressed genes; DEGs, differentially expressed genes; LAMRGs, lactic acid metabolism-related genes; DEGs, differentially expressed genes; TCGA, The Cancer Genome Atlas; LIHC, liver hepatocellular carcinoma.

The flow chart of this study is presented in Figure 1. The data from TCGA-LIHC dataset were divided into HCC and normal groups. Ninety-four DEGs between the two groups were identified using the limma package. The filtering threshold was set at |logFC| > 2 and *p*. adjust< 0.05. There were 15 upregulated and 79 downregulated genes (Figures 2A, B). The LAMRGs were obtained from the GeneCards database and literature. To obtain LAMRDEGs, we intersected genes from the LAMRGs and the above DEGs. We then identified five upregulated (CCNB1, E2F1, NQO1,LCN2,SPP1) and 23 downregulated (ADAMTS13, ALPL, BCHE, CFP, CXCL12, CYP2A6, CYP2C9, DBH, FBP1, FOS, GPD1, GYS2, HBB, HP, IGF2, IGFBP3, KDM8, LCAT, PCK1, RCAN1, SERPINE1, SHBG, and TDO2) LAMRDEGs.

The venndiagram and heatmap were constructed to illustrate the LAMRDEGs between the HCC and normal tissues (Figures 2C–E).

### Function enrichment analysis of LAMRDEGs

**FIGURE 3:**
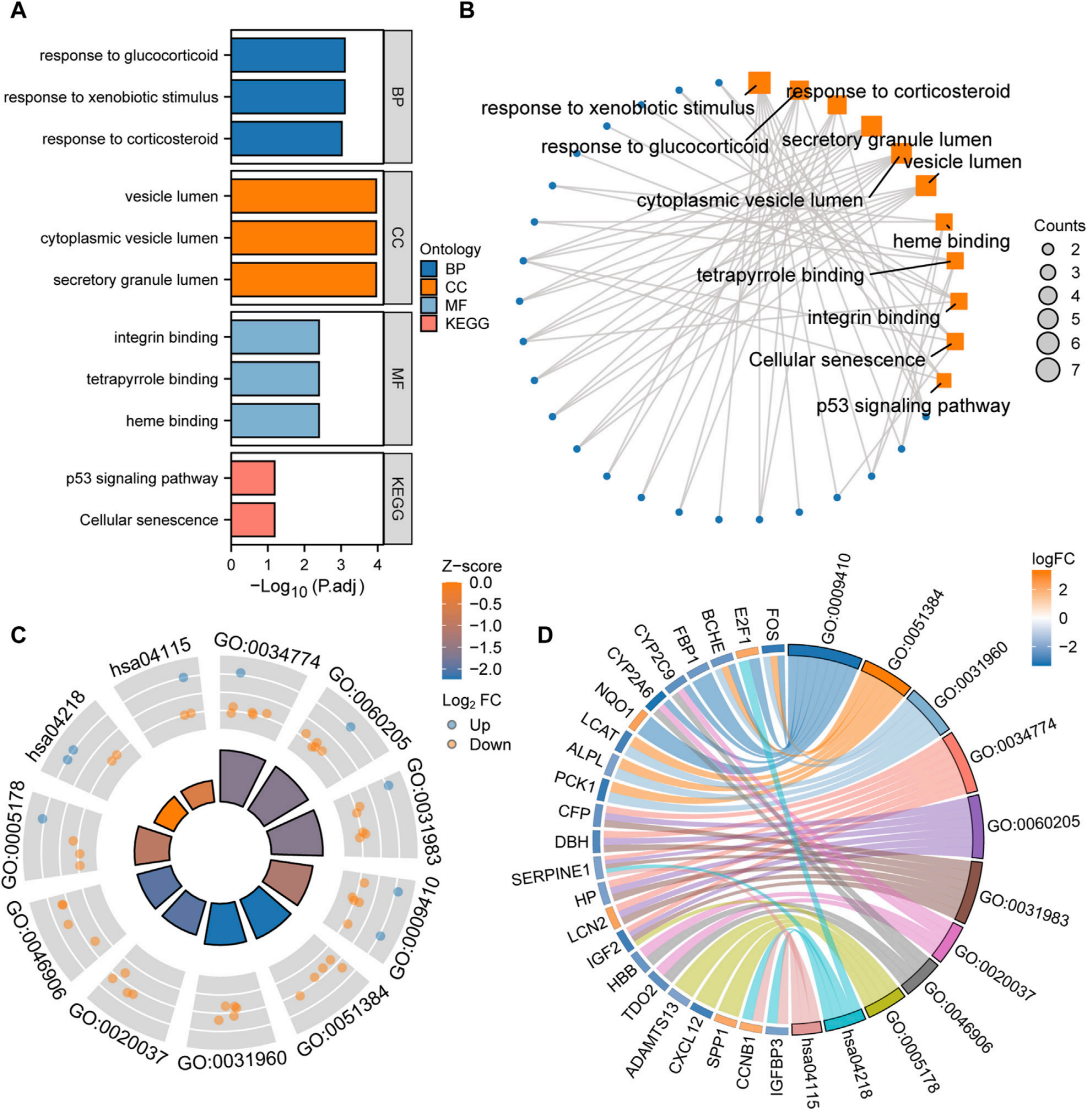
GO and KEGG enrichment analysis of the LAMRDEGs **(A)**. Histogram of the GO and KEGG analyses results regarding the LAMRDEGs. **(B)**. Network map of the GO and KEGG analyses results regarding the LAMRDEGs. **(C)**. Cyclic graph of the combined logFC values and GO and KEGG analyses results regarding the LAMRDEGs. **(D)**. Chord plot of the combined logFC and GO and KEGG analyses results regarding the LAMRDEGs. LAMRDEGs, lactic acid metabolism-related differentially expressed genes; GO, Gene Ontology; BP, biological process; CC, cellular component; MF, molecular function; KEGG, Kyoto Encyclopedia of Genes and Genomes.

GO and KEGG enrichment analyses of the LAMRDEGs were conducted. The results are illustrated in Figures 3A, B. Subsequently, we conducted GO and KEGG enrichment analyses of the LAMRDEGs combined with the logFC values. The results are presented in Figures 3C, D. As shown in Figure 3A-D, the LAMRDEGs were mainly enriched in biological processes, including response to xenobiotic stimulus (GO, 0009410), response to glucocorticoid (GO, 0051384), response to corticosteroid (GO, 0031960). The LAMRDEGs were significantly enriched in the secretory granule lumen (GO, 0034774), cytoplasmic vesicle lumen (GO, 0060205), and vesicle lumen (GO, 0031983) of GO cellular components (CC). The LAMRDEGs were also enriched in heme binding (GO, 0020037), tetrapyrrole binding (GO, 0046906), integrin binding (GO, 0005178) of molecular functions (MF). In addition, the KEGG pathway analysis showed that the pathways were enriched in cellular senescence (hsa04218), and the p53 signaling pathway (hsa04115) (Table 1).

**TABLE 1:** GO and KEGG enrichment analysis results of DEGs.

Ontology	ID	Description	GeneRatio	BgRatio	*p*-value	P.adj
BP	GO, 0009410	response to xenobiotic stimulus	7/27	411/18800	1.38e-06	0.0008
BP	GO, 0051384	response to glucocorticoid	5/27	139/18800	1.46e-06	0.0008
BP	GO, 0031960	response to corticosteroid	5/27	157/18800	2.65e-06	0.0010
BP	GO, 0071466	cellular response to xenobiotic stimulus	5/27	168/18800	3.7e-06	0.0010
BP	GO, 0034637	cellular carbohydrate biosynthetic process	4/27	79/18800	4.71e-06	0.0010
CC	GO, 0034774	secretory granule lumen	6/28	322/19594	5.22e-06	0.0001
CC	GO, 0060205	cytoplasmic vesicle lumen	6/28	325/19594	5.51e-06	0.0001
CC	GO, 0031983	vesicle lumen	6/28	327/19594	5.7e-06	0.0001
CC	GO, 1904724	tertiary granule lumen	3/28	55/19594	6.52e-05	0.0008
CC	GO, 0005788	endoplasmic reticulum lumen	5/28	311/19594	7.1e-05	0.0008
MF	GO, 0020037	heme binding	4/28	139/18410	5.53e-05	0.0040
MF	GO, 0046906	tetrapyrrole binding	4/28	149/18410	7.25e-05	0.0040
MF	GO, 0005178	integrin binding	4/28	156/18410	8.67e-05	0.0040
MF	GO, 0016705	oxidoreductase activity, acting on paired donors, with incorporation or reduction of molecular oxygen	4/28	177/18410	0.0001	0.0049
MF	GO, 0016209	antioxidant activity	3/28	85/18410	0.0003	0.0069
KEGG	hsa04218	Cellular senescence	4/23	156/8164	0.0009	0.0652
KEGG	hsa04115	p53 signaling pathway	3/23	73/8164	0.0011	0.0652

GO, gene ontology; BP, biological process; CC, cellular component; MF, molecular function; KEGG, kyoto encyclopedia of genes and genomes; DEGs, Differentially expressed genes.

### GSEA of DEGs

**FIGURE 4:**
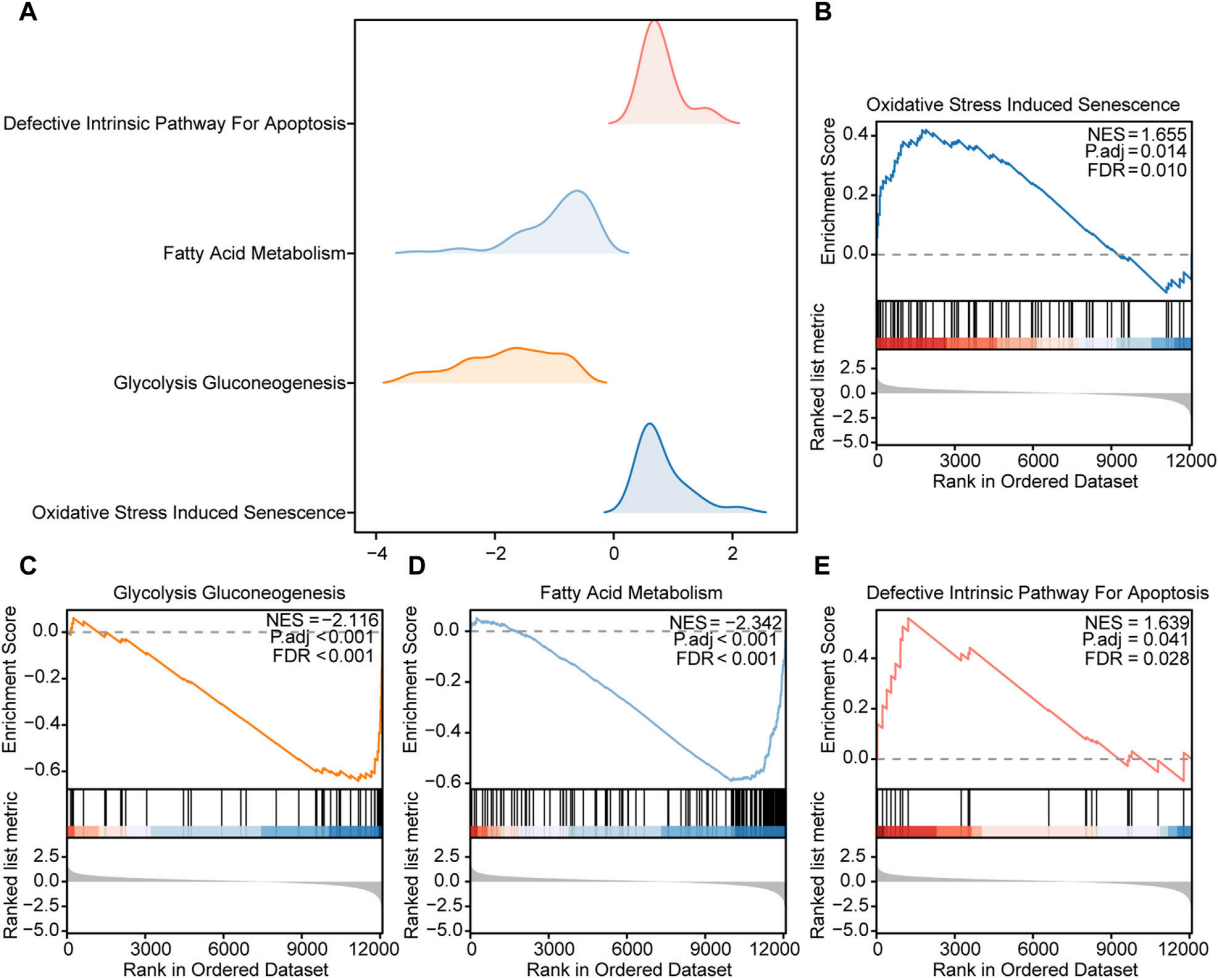
GSEA of the DEGs **(A)**. Ridgeline plot of the four main biological functions of GSEA in TCGA-LIHC dataset. B–E. The DEGs in TCGA-LIHC dataset were significantly enriched in pathways including **(B)** oxidative stress-induced senescence, **(C)** glycolysis gluconeogenesis, **(D)** fatty acid metabolism, and **(E)** the defective intrinsic pathway for apoptosis.

To further elucidate the potential mechanisms of the DEGs in HCC, we used GSEA to explore prominent KEGG pathways in the expression of DEGs from TCGA-LIHC dataset. The enrichment results are presented as a ridgelineplot (Figure 4A). The results showed that the DEGs were significantly enriched in different pathways (Table 2), including oxidative stress induced senescence (Figure 4B), glycolysis gluconeogenesis (Figure 4C), fatty acid metabolism (Figure 4D), and the defective intrinsic pathway for apoptosis (Figure 4E).

**TABLE 2:** GSEA analysis of TCGA-LIHC.

ID	SetSize	EnrichmentScore	NES	*p*-value	P.adj	Qvalue
KEGG_RIBOSOME	85	0.7611006	3.085902	1e-10	4.38e-09	3.03e-09
WP_CYTOPLASMIC_RIBOSOMAL_PROTEINS	86	0.7587952	3.078273	1e-10	4.38e-09	3.03e-09
REACTOME_INFLUENZA_INFECTION	151	0.6979987	3.075782	1e-10	4.38e-09	3.03e-09
REACTOME_EUKARYOTIC_TRANSLATION_ELONGATION	89	0.7507659	3.034779	1e-10	4.38e-09	3.03e-09
REACTOME_SRP_DEPENDENT_COTRANSLATIONAL_PROTEIN_TARGETING_TO_MEMBRANE	109	0.7127232	2.974571	1e-10	4.38e-09	3.03e-09
REACTOME_EUKARYOTIC_TRANSLATION_INITIATION	116	0.6987621	2.945497	1e-10	4.38e-09	3.03e-09
REACTOME_NONSENSE_MEDIATED_DECAY_NMD	112	0.6940664	2.910544	1e-10	4.38e-09	3.03e-09
REACTOME_RESPONSE_OF_EIF2AK4_GCN2_TO_AMINO_ACID_DEFICIENCY	98	0.6981658	2.869411	1e-10	4.38e-09	3.03e-09
REACTOME_REGULATION_OF_EXPRESSION_OF_SLITS_AND_ROBOS	155	0.6393451	2.824605	1e-10	4.38e-09	3.03e-09
REACTOME_CELL_CYCLE_MITOTIC	478	0.5605160	2.806142	1e-10	4.38e-09	3.03e-09

GSEA, gene set enrichment analysis.

### Cox Model for prognostic prediction

**FIGURE 5:**
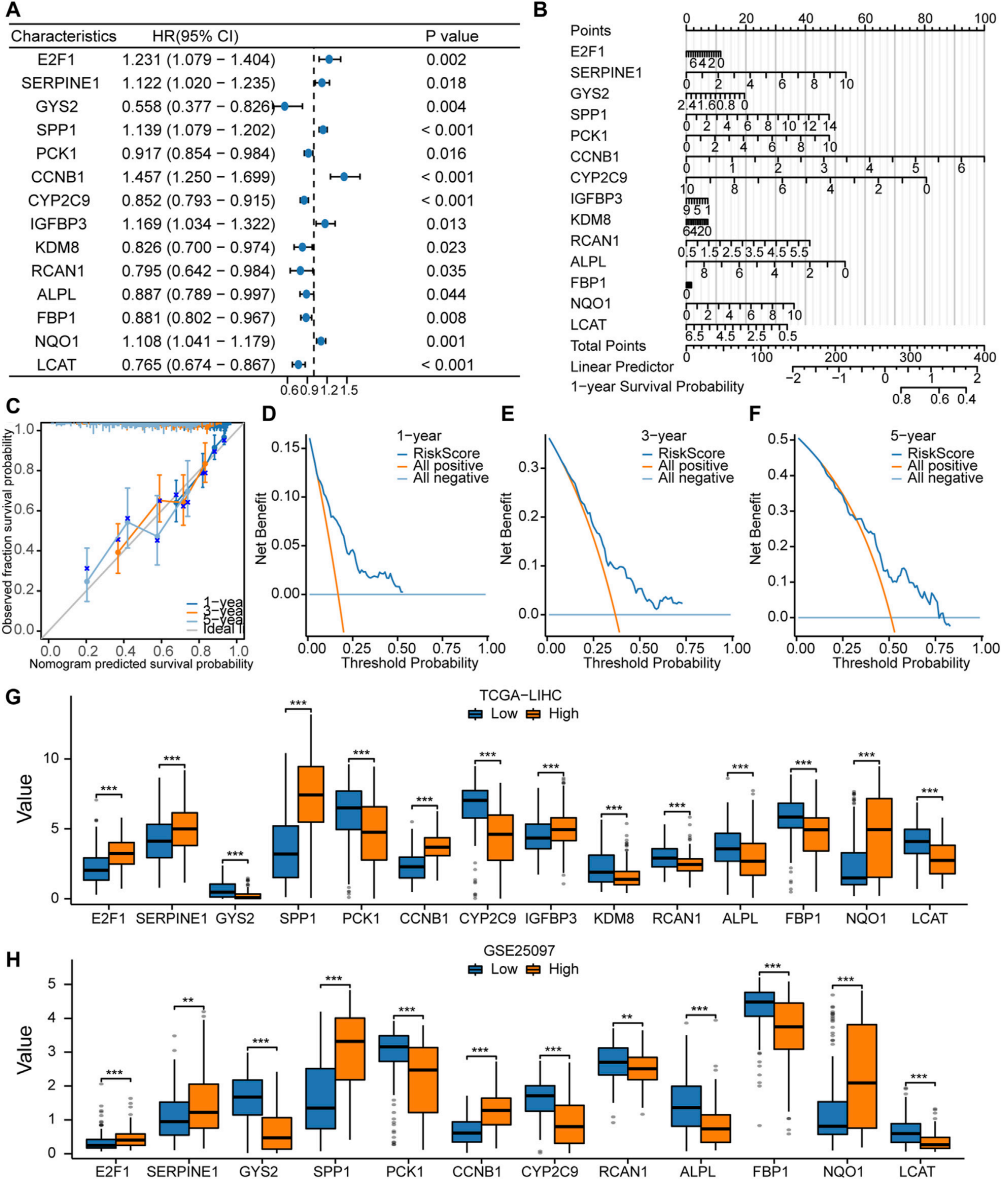
Constructionof the Cox regression model **(A, B)**. Forest plot **(A)** and nomogram **(B)** regarding the univariate and multivariate Cox regression analyses of the key genes. **(C)**. Calibration curve plots for the 1-, 3-, and 5-year survival using the Cox regression model based on the nomogram analysis. **(D–F)**. DCA regarding the 1-, 3-, and 5-year survival based on the Cox regression model. **(G)**. Comparison of the expression levels of the 14 key genes between the high- and low-risk groups in TCGA-LIHC dataset. **(H)**. Comparison of the expression levels of the 12 key genes between the high- and low-risk groups in the GSE25097 dataset.

The univariate Cox regression analysis was used to assess the prognostic value of LAMRDEGs. The screening revealed that 14 key genes (E2F1, SERPINE1, GYS2, SPP1, PCK1, CCNB1, CYP2C9, IGFBP3, KDM8, RCAN1, ALPL, FBP1, NQO1, and LCAT) were associated with the over survival (Table 3; Figure 5A). We constructed a nomogram to assess the predictive value of the Cox model using these genes (Figure 5B). In addition, we constructed 1-year, 3-year, and 5-year (Figure 5C) calibration curves regarding nomogram. The results indicated that the prediction effect of the model in the 5-year survival analysis was better than that in the 1-year and 3-year survival analyses. Moreover, we used the DCA to evaluate the clinical utility of the prognostic model in terms of the 1-year (Figure 5D), 3-year (Figure 5E), and 5-year (Figure 5F). The results suggested that the prediction effect of the model in the 5-year survival analysis was better than that in 1- and 3-year survival analyses.

**TABLE 3:** Cox regression of dataset TCGA-LIHC.

Characteristics	Total (N)	Univariate analysis		Multivariate analysis	
		Hazard ratio (95% CI)	*p*-value	Hazard ratio (95% CI)	*p*-value
E2F1	373	1.231 (1.079–1.404)	0.002	0.975 (0.781–1.218)	0.827
SERPINE1	373	1.122 (1.020–1.235)	0.018	1.090 (0.966–1.231)	0.163
GYS2	373	0.558 (0.377–0.826)	0.004	0.877 (0.533–1.444)	0.606
SPP1	373	1.139 (1.079–1.202)	<0.001	1.057 (0.986–1.133)	0.119
PCK1	373	0.917 (0.854–0.984)	0.016	1.081 (0.972–1.202)	0.153
CCNB1	373	1.457 (1.250–1.699)	<0.001	1.282 (0.989–1.662)	0.061
CYP2C9	373	0.852 (0.793–0.915)	<0.001	0.878 (0.794–0.971)	**0.011**
IGFBP3	373	1.169 (1.034–1.322)	0.013	0.986 (0.848–1.145)	0.848
KDM8	373	0.826 (0.700–0.974)	0.023	0.981 (0.794–1.211)	0.857
RCAN1	373	0.795 (0.642–0.984)	0.035	1.129 (0.865–1.475)	0.371
ALPL	373	0.887 (0.789–0.997)	0.044	0.909 (0.790–1.045)	0.181
FBP1	373	0.881 (0.802–0.967)	0.008	1.003 (0.879–1.145)	0.963
NQO1	373	1.108 (1.041–1.179)	0.001	1.060 (0.983–1.143)	0.128
LCAT	373	0.765 (0.674–0.867)	<0.001	0.919 (0.785–1.077)	0.296

TCGA, the cancer genome atlas; LIHC, liver hepatocellular carcinoma.

The meaning of bold value was that multivariate analysis showed *P* <0.05.

In addition, we divided TCGA-LIHC and GSE25097 samples into high- and low-risk groups according to the risk scores and compared expression of 14 key genes between the high- and low-risk groups from TCGA-LIHC dataset (Figure 5G). In the GSE25097 dataset, only the expression levels of 12 key genes (E2F1, SERPINE1, GYS2, SPP1, PCK1, CCNB1, CYP2C9, RCAN1, ALPL, FBP1, NQO1, and LCAT) differed significantly between the high- and low-risk groups (Figure 5H).

### Gene Set Variation Analysis

**FIGURE 6:**
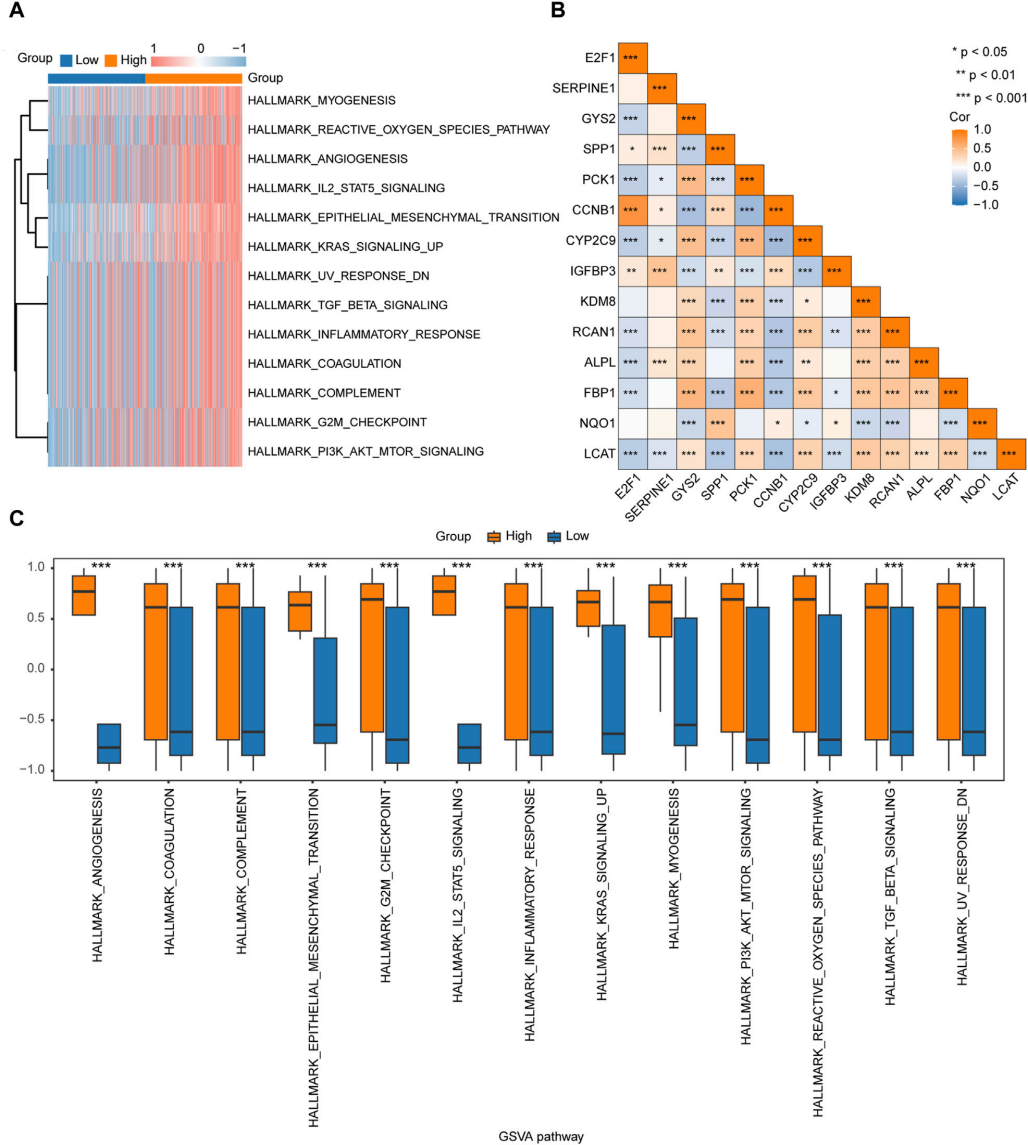
GSVA of TCGA-LIHC dataset **(A)**. Heatmap of the functional scores in the GSVA. **(B)**. Heatmap of the correlation between the 14 key genes. **(C)**. Comparison of the significant enrichment pathways in the GSVA between the high- and low-risk groups.

To explore the differences in the hallmark gene set between the high- and low-risk groups, GSVA was performed on TCGA-LIHC dataset. The results showed that different genes between the high- and low-risk groups in the LIHC samples were significantly enriched in 17 pathways, of which 13 were hallmark gene sets (angiogenesis, coagulation, complement, epithelial mesenchymal transition, G2M checkpoint, IL2/STAT5 signaling, inflammatory response, KRAS signaling up, myogenesis, PI3K/AKT/mTOR signaling, reactive oxygen species pathway, TGF-β signaling, and UV response DN) (Figure 6A; Table 4). A comparison of the hallmark gene sets is presented in Figure 6C. A correlation heatmap of the 14 key genes is presented in Figure 6B.

**TABLE 4:** GSVA analysis of TCGA-LIHC dataset.

Ontology	logFC	AveExpr	t	P.Value	adj.P
HALLMARK_ANGIOGENESIS	−0.907354821	−0.010311404	−12.99538088	9.37E-33	7.97E-32
HALLMARK_IL2_STAT5_SIGNALING	−0.907354821	−0.010311404	−12.99538088	9.37E-33	7.97E-32
HALLMARK_EPITHELIAL_MESENCHYMAL_TRANSITION	−0.751514215	0.038111566	−12.74604327	9.58E-32	5.43E-31
HALLMARK_KRAS_SIGNALING_UP	−0.756255157	0.048359743	−11.90858684	2.02E-28	8.60E-28
HALLMARK_MYOGENESIS	−0.595822385	0.072268378	−9.040133663	5.56E-18	1.89E-17
HALLMARK_G2M_CHECKPOINT	−0.601469372	0.01938544	−7.833225589	3.83E-14	9.30E-14
HALLMARK_PI3K_AKT_MTOR_SIGNALING	−0.601469372	0.01938544	−7.833225589	3.83E-14	9.30E-14
HALLMARK_REACTIVE_OXYGEN_SPECIES_PATHWAY	−0.585543805	−0.027634564	−7.596308483	1.95E-13	4.15E-13
HALLMARK_TGF_BETA_SIGNALING	−0.415745545	−0.002474737	−5.284238664	2.02E-07	2.64E-07
HALLMARK_COMPLEMENT	−0.415745545	−0.002474737	−5.284238664	2.02E-07	2.64E-07
HALLMARK_INFLAMMATORY_RESPONSE	−0.415745545	−0.002474737	−5.284238664	2.02E-07	2.64E-07
HALLMARK_UV_RESPONSE_DN	−0.415745545	−0.002474737	−5.284238664	2.02E-07	2.64E-07
HALLMARK_COAGULATION	−0.415745545	−0.002474737	−5.284238664	2.02E-07	2.64E-07

GSVA, gene set variation analysis; TCGAthe, cancer genome atlas; LIHC, liver hepatocellular carcinoma.

### Construction of the predictive model based on LASSO regression

**FIGURE 7:**
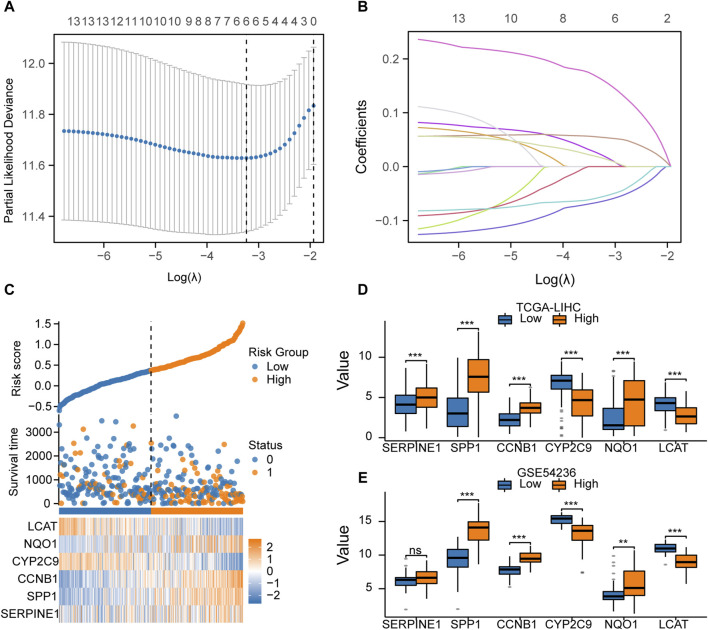
Construction of the prognostic model based on LASSO regression **(A)**. LASSO regression model with 14 key genes to screen the prognosis-related genes. **(B)**. LASSO regression was performed to screen out the six key genes. **(C)**. Risk score, risk grouping, survival status, and expression of the six key genes in TCGA-LIHC dataset. **(D)**. Comparison of the expression of the six key genes between the high- and low-risk groups in TCGA-LIHC dataset. **(E)**. Comparison of the expression of the six key genes between the high- and low-risk groups in theGSE54236dataset.

We screened key prognosis-related genes using LASSO regression (Figures 7A, B) and constructed a predictive model for prognosis. The prognostic model comprised six key genes: SERPINE1, SPP1, CCNB1, CYP2C9, NQO1, and LCAT (Table 5). A risk scoreplot for the six key genes is illustrated in Figure 7C. Finally, according to the risk score of the predictive model, the samples from TCGA-LIHC and GSE54236 datasets were divided into high- and low-risk groups. The expression levels of the six key genes between the high-and low-risk groups from TCGA-LIHC (Figure 7D) and GSE54236 (Figure 7E) were compared.

**TABLE 5:** List of key genes of differential expression analysis.

Gene Symbol	Description	logFC	P.Value	adj.P.Val
LCAT	lecithin-cholesterol acyltransferase	−2.83466	3.36E-35	6.10E-33
CCNB1	cyclin B1	2.024582	2.36E-32	3.26E-30
SERPINE1	serpin family E member 1	−2.05142	7.84E-15	7.11E-14
CYP2C9	cytochrome P450 family 2 subfamily C member 9	−2.45995	1.48E-13	1.11E-12
NQO1	NAD (P) H quinone dehydrogenase 1	2.293504	3.02E-09	1.22E-08
SPP1	secreted phosphoprotein 1	2.092279	9.64E-06	2.36E-05

### Prognostic value of the key genes for HCC

**FIGURE 8:**
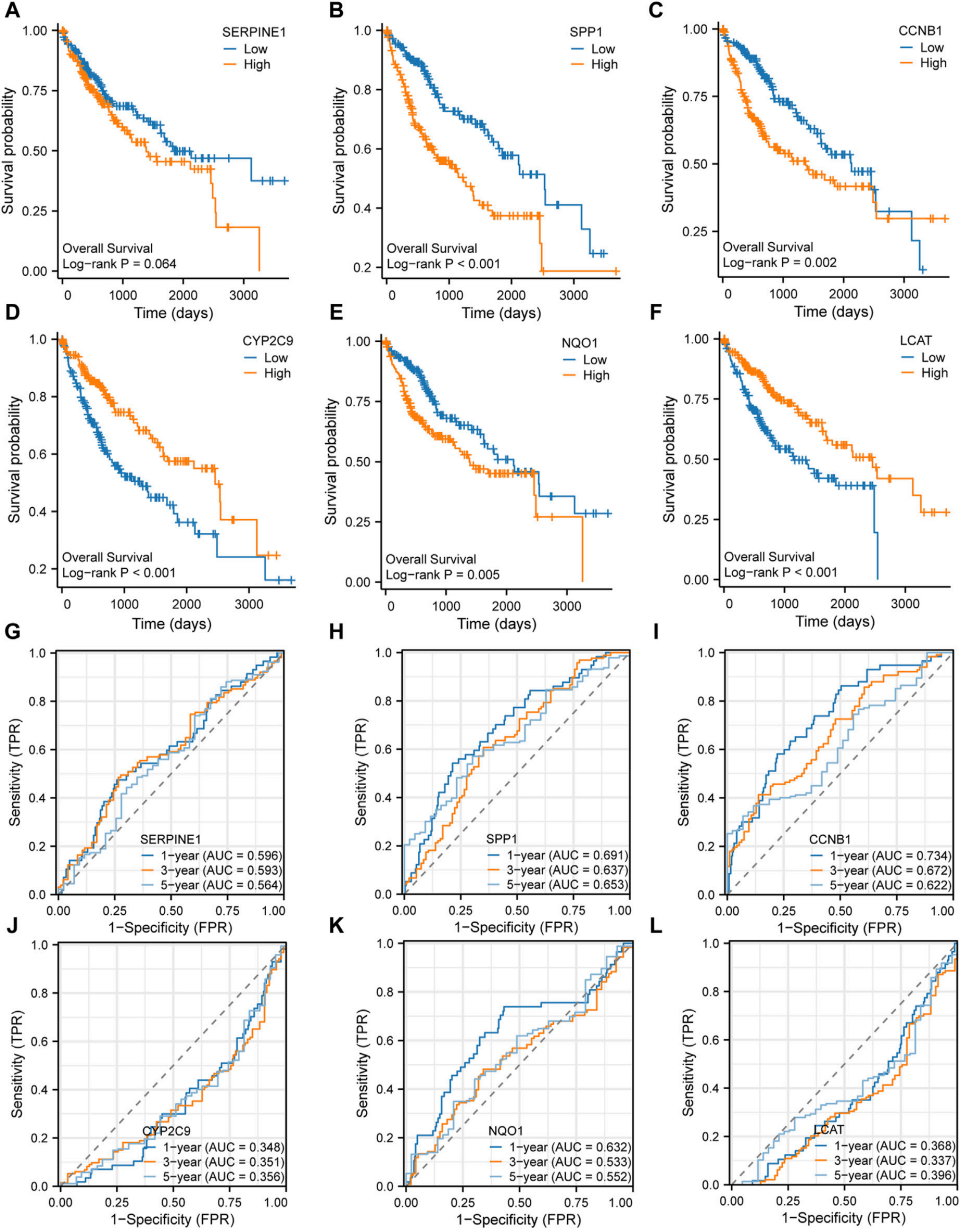
Prognosis analysis of the six key genes **(A–F)**. Kaplan-Meier survival analysis of the six key genes **(G–L)**. Time-dependent receiver operating characteristic (ROC) analysis of the six key genes.

The Kaplan-Meier survival analysis was performed to examine the prognostic value of the six key genes (SERPINE1, SPP1, CCNB1, CYP2C9, NQO1, and LCAT) in the high expression and low-expression groups (Figures 8A–F). The results showed that the high expression of SERPINE1 (Figure 8A), SPP1 (Figure 8B), CCNB1 (Figure 8C) and NQO1 (Figure 8E) and low expression of CYP2C9 (Figure 8D) and LCAT (Figure 8F) were associated with poor prognosis. In addition, 1-, 3- and 5-year time-dependent receiver operating characteristic curves were plotted to analyze the prognostic value of the six key genes in TCGA-LIHC dataset. The results showed that the expression levels of SERPINE1 (Figure 8G), SPP1 (Figure 8H), CCNB1 (Figure 8I), and NQO1 (Figure 8K) had prognostic predictive value in HCC patients, while CYP2C9 (Figure 8J) and LCAT (Figure 8L) had no significant prognostic value.

### Construction of the PPI networks

**FIGURE 9:**
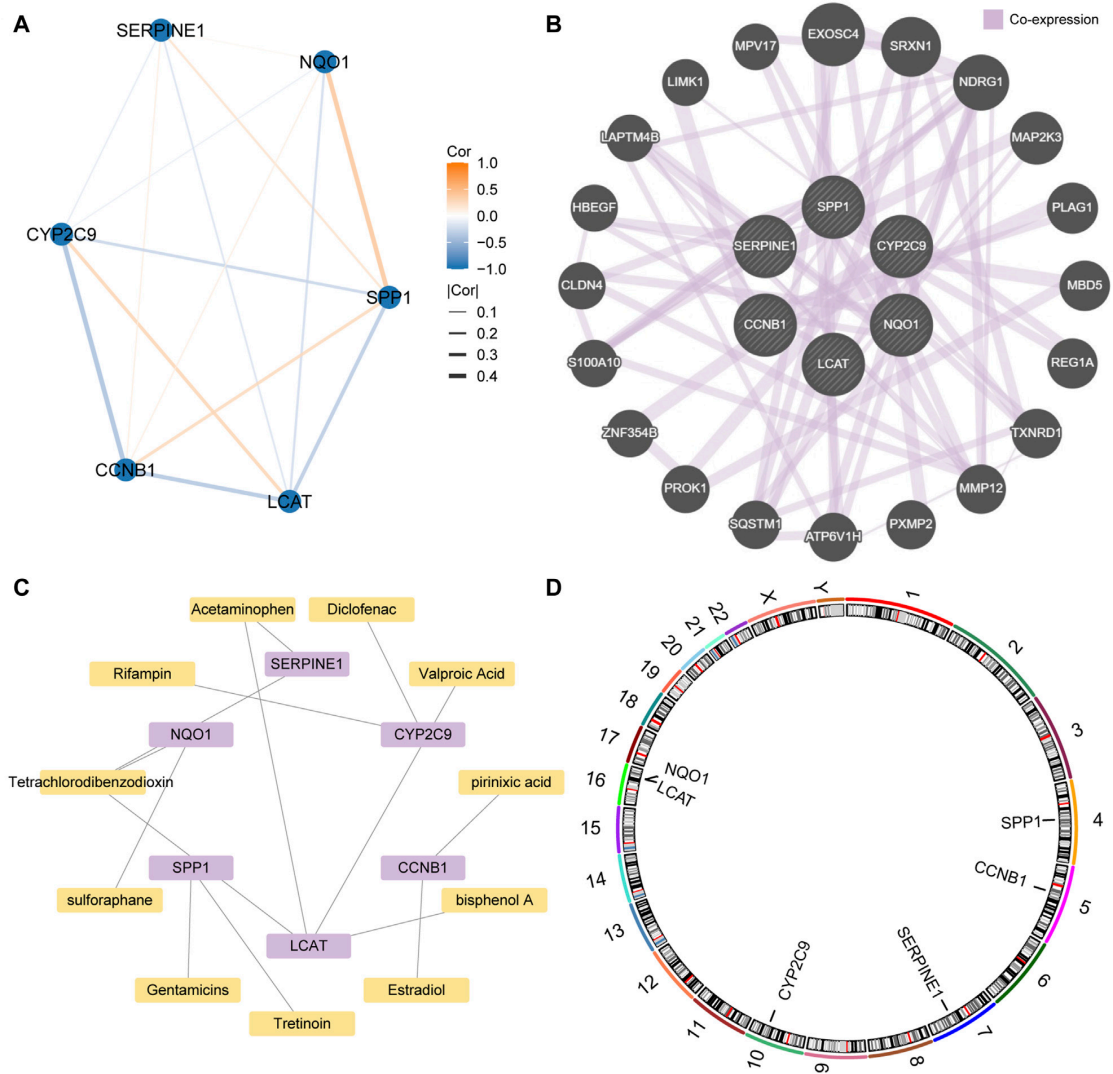
Protein-protein interaction (PPI) network **(A)**. PPI network of the six key genes. **(B)**. The interaction network of the predicted functionally similar genes among the six key genes **(C)**. Interaction network of the mRNA-based and small molecule drugs among the six key genes. **(D)**. Chromosome mapping of the six key genes.

Since genes that regulate the same biological processes have close relationships, we further analyzed the interaction between the key genes. The STRING database was used to construct a PPI network of the six key genes (SERPINE1, SPP1, CCNB1, CYP2C9, NQO1, and LCAT) (Figure 9A). We found that NQO1 had the strongest positive correlation with SPP1, CYP2C9 had the strongest negative correlation with CCNB1, and SERPINE1 had the weakest correlation with the other genes. In addition, we constructed and predicted the functionally similar gene interaction network of six key genes using GeneMANIA and found that 20 genes were co-expressed with the six key genes (Figure 9B).As shown in the mRNA-drug interaction network constructed using the CTD database (Figure 9C), we identified 11 potential drugs or compounds for the six key genes.

Finally, chromosomal mapping was performed regarding the six key genes. The results showed that SPP1 was distributed on chromosome 4, CCNB1 on chromosome 5, SERPINE1 on chromosome 7, and CYP2C9 on chromosome 10. LCAT and NQO1 were distributed on chromosome 16 (Figure 9D).

### Immune infiltrate analysis

**FIGURE 10:**
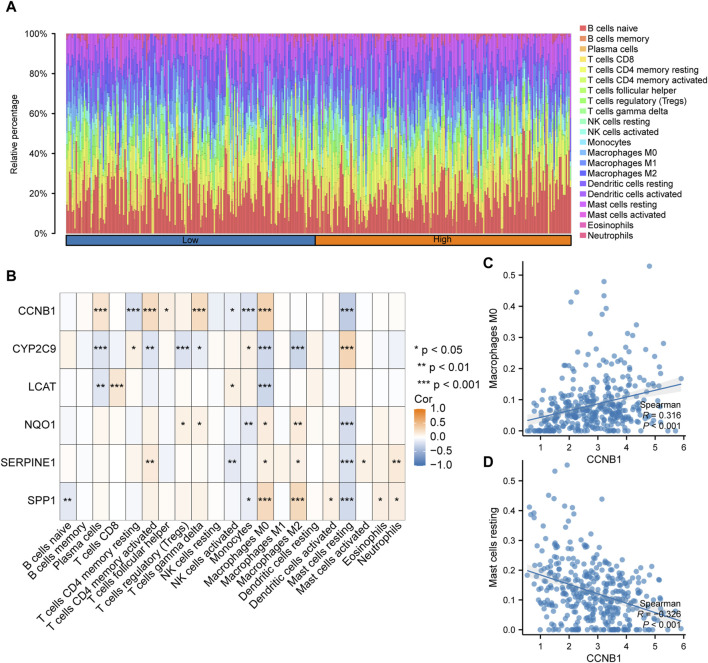
Analysis of immune cell infiltration based on the risk scores **(A)**. Histogram of the proportion of immune cells among the different samples in TCGA-LIHC dataset. **(B)**. Correlation between the immune cell infiltration abundance and six key genes in TCGA-LIHC dataset. **(C)**. CCNB1 was positively correlated with macrophages M0. **(D)**. CCNB1was negatively correlated with resting mast cells.

The expression profile data and grouping information were collated and uploaded to the CIBERSORTx. The correlation between the 22 types of immune cells and the expression profiles of the high- and low-risk group samples were assessed. According to the analysis results, the distribution of immune cell infiltration in TCGA-LIHC dataset is illustrated in Figure 10A **.** The high-risk group had a higher abundance of macrophage, CD4^+^ memory active T cells and gamma-delta T cells (γδT), and had a lower abundance of mast cells. In addition, the correlation between immune cell infiltration abundance and the six key genes was analyzed, and the correlation heatmap is illustrated in Figure 10B.The positive correlation between CCNB1 and macrophages M0 was the top1 (*p* < 0.001; r = 0.316; Figure 10C). The negative correlation between CCNB1 and mast cells resting was the top1 (*p* < 0.001; r = −0.326; Figure 10D).

## Discussion

Increasing evidence indicates that lactic acid metabolic changes in tumors can remodel the TME and confer a growth advantage to tumor cells (Romero-Garcia et al., 2016; Gu et al., 2022). Previous studies have indicated lactic acid promotes the proliferation, invasion, and migration of HCC cells (Zeng et al., 2020). In recent years, the inhibition of lactate metabolism has proven to be a potential therapeutic approach for malignant tumors (Doherty and Cleveland, 2013). It has been reported that the simultaneous inhibition of lactate metabolism related gene ODC1 and PKM2 exerts synergistic effects against HCC cells (Zeng et al., 2020). Recently, a prediction model of lactic acid metabolism-related lncRNAs was constructed and used to predict the clinical outcomes and assess the TME in patients with HCC (Guan et al., 2022). Therefore, it is necessary to understand the functions and mechanisms of LAMRGs in the development of HCC.

In this study, LAMRDEGs were screened out and their functions and pathways were enriched using GO, KEGG, and GSEA. Finally, a model of six genes that could predict the prognosis of patients with HCC was constructed. These LAMRDEGs, including CCNB1, CYP2C9, E2F1, NQO1, LCAT, SERPINE1, and SPP1, may be involved in lactic acid transport, production, and consumption. Abnormal expressions of these genes lead to an acidified microenvironment, thereby affecting the occurrence, development, metastasis, and progression of HCC. A previous study has reported that SERPINE1 facilitated HCC progression (Li et al., 2021). SPP1 and CYP2C9 have also been as prognostic biomarkers for HCC (Tu et al., 2023). CCNB1 can promote cell proliferation, migration, invasion and resistance in HCC (Gu et al., 2019; Jin et al., 2020; Xia et al., 2021). Reducing CCNB1 expression can suppresses the growth of HCC cells (Li et al., 2021). LCAT has been reported as a prognostic and recurrence biomarker in HCC (Long et al., 2019; Hu et al., 2020; Jiang et al., 2020; Su et al., 2021; Sharifi et al., 2022) and has been correlated to anti-cancer drug sensitivities (Zhang et al., 2022). Collectively, LAMRDEGs may serves as prognostic biomarkers and potential targets for the development of new therapeutic strategies for HCC.

Moreover, our results indicated that LAMRDEGs were involved in lactate metabolism and HCC related pathways: oxidoreductase activity, incorporation or reduction of molecular oxygen, antioxidant activity, cellular senescence, and p53 signaling pathways. High oxidoreductase activity can promote HCC carcinogenesis, targeting oxidoreductase activity-related pathways can be a potential treatment modality for human cancer (Kudo et al., 2014). The incorporation or reduction of molecular oxygen and powerful antioxidant activity play a key role in HCC treatment (Ahmed et al., 2018; Kim et al., 2022). In addition, cellular senescence affects immune infiltration and immunotherapeutic responses in HCC (Gao et al., 2023). The activation of the P53 signaling pathway is involved in HCC carcinogenesis, and inhibition of P53 can improve the therapies response in HCC (Krstic et al., 2022). Therefore, LAMRDEGs are involved in the development and progression of HCC.

In addition, the GSEA indicated that the LAMRDEGs were mainly enriched in cancer associated pathways including oxidative stress-induced senescence, glycolysis, gluconeogenesis, fatty acid metabolism, and the defective intrinsic pathway for apoptosis (Hanahan, 2022). Moreover, the GSVA showed that the hallmark gene sets such as angiogenesis, epithelial mesenchymal transition, G2M checkpoint, KRAS signaling up, PI3K/AKT/mTOR signaling, and TGF-β signaling exhibited significant differences between high- and low-risk groups in terms of the expression patterns of LAMRDEGs. These different gene sets are closely related to tumor cells proliferation, invasion, and metastasis (Dituri et al., 2019; Satyananda et al., 2021; Li et al., 2022; Wu et al., 2022; Han et al., 2023). Based on these results, we speculate that these LAMRDEGs are involved in the progression of HCC and have potential applications in evaluating its prognosis.

Lactic acid is essential energy substances for tumor metabolism and play an indispensable role in restructuring the TME (Bergers and Fendt, 2021; Hayes et al., 2021). It is also an immunosuppressive molecule in immune infiltration of TME. On the one hand, the high lactic acid levels in TME lead to decrease in the production of T cell related cytokines and T cell activity, and can inhibit the survival and function of NK cells, associating with decreased immune infiltration and aggressive tumor progression (Pérez-Tomás and Pérez-Guillén, 2020; Watson et al., 2021). On the other hand, high lactic acid levels derived from TME can promote the M2 polarization of tumor-associated macrophages (TAM), and assist TAM in promoting tumor growth (Goswami et al., 2022). The abnormal expressions of these LAMRDEGs are associated with tumor immune escape and affect the efficacy of therapy. The SERPINE1 is an immune infiltration related gene in several cancers (Wang et al., 2023; Zhai et al., 2023). Targeting the E2F1/SEC61G pathway increased response to chemotherapy in hypopharyngeal squamous cell carcinoma through immune response (Li et al., 2022). High CCNB1expression is associated with tumor immune infiltration and poor prognosis in breast cancer (Fu et al., 2022). Combining NQO1 inhibitors with conventional chemotherapeutics might enhance anti-tumor immune effects in non-small cell lung cancer (Madajewski et al., 2023). Furthermore, lower LCAT expression and higher infiltration of immune cells have been detected in patients with HCC (Hu et al., 2020). Our findings indicated that the expression of LAMRDEGs was associated with immune cell types in high risk groups. Moreover, key genes were significantly correlated with cell immune infiltrating levels, such as those regarding T cells, and macrophages. Hence, these immune cells infiltration might promote tumor progression by suppressing anti-tumor immunity in the high-risk group. These results suggest that the LAMRDEGs can play a crucial role in the regulation of immune cell infiltration and might be potential prognosis biomarkers in patients with HCC. However, whether these key genes can be therapeutic target in HCC required further investigation.

This study had some limitations. First, no further experimental validation was conducted due to limited experiment funding and sample availability. Second, the insufficient sample size may have affected the reliability of results. Further prospective, multicenter studies are needed to evaluate clinical value. Third, there are few reports related to lactate metabolism in HCC of these hub genes, further research into their mechanisms is required.

In summary, we explored the functions and potential mechanisms associated with LAMRDEGs in HCC. Furthermore, we constructed a prognostic model using LAMRDEGs. Our findings may provide new biomarkers for evaluating the survival outcomes of patients with HCC and new insights into the potential therapeutic targets for HCC. However, the specific pathological mechanisms and molecular targets in HCC require further investigation. Further studies with experimental validations and larger sample sizes are needed to strengthen the veracity of our findings.

## Data Availability

The datasets presented in this study can be found in online repositories. The names of the repository/repositories and accession number(s) can be found below: https://www.ncbi.nlm.nih.gov/geo/, GSE25097. https://www.ncbi.nlm.nih.gov/geo/, GSE54236. https://portal.gdc.cancer.gov/projects/TCGA-LIHC, TCGA-LIHC.
