# Supplementary material for: Identification and functional analysis of lactic acid metabolism-related differentially expressed genes in hepatocellular carcinoma
Source: Front Genet. 2024 Apr 16;15:1390882. doi: 10.3389/fgene.2024.1390882 (PMC11058226; doi:10.3389/fgene.2024.1390882)
Supplement: Supplementary file 2 [file Table1.DOCX]

**Table1. GO and KEGG enrichment analysis results of DEGs**

| Ontology | ID | Description | GeneRatio | BgRatio | Pvalue | P.adj |
| --- | --- | --- | --- | --- | --- | --- |
| BP | GO，0009410 | response to xenobiotic stimulus | 7/27 | 411/18800 | 1.38e-06 | 0.0008 |
| BP | GO，0051384 | response to glucocorticoid | 5/27 | 139/18800 | 1.46e-06 | 0.0008 |
| BP | GO，0031960 | response to corticosteroid | 5/27 | 157/18800 | 2.65e-06 | 0.0010 |
| BP | GO，0071466 | cellular response to xenobiotic stimulus | 5/27 | 168/18800 | 3.7e-06 | 0.0010 |
| BP | GO，0034637 | cellular carbohydrate biosynthetic process | 4/27 | 79/18800 | 4.71e-06 | 0.0010 |
| CC | GO，0034774 | secretory granule lumen | 6/28 | 322/19594 | 5.22e-06 | 0.0001 |
| CC | GO，0060205 | cytoplasmic vesicle lumen | 6/28 | 325/19594 | 5.51e-06 | 0.0001 |
| CC | GO，0031983 | vesicle lumen | 6/28 | 327/19594 | 5.7e-06 | 0.0001 |
| CC | GO，1904724 | tertiary granule lumen | 3/28 | 55/19594 | 6.52e-05 | 0.0008 |
| CC | GO，0005788 | endoplasmic reticulum lumen | 5/28 | 311/19594 | 7.1e-05 | 0.0008 |
| MF | GO，0020037 | heme binding | 4/28 | 139/18410 | 5.53e-05 | 0.0040 |
| MF | GO，0046906 | tetrapyrrole binding | 4/28 | 149/18410 | 7.25e-05 | 0.0040 |
| MF | GO，0005178 | integrin binding | 4/28 | 156/18410 | 8.67e-05 | 0.0040 |
| MF | GO，0016705 | oxidoreductase activity, acting on paired donors， with incorporation or reduction of molecular oxygen | 4/28 | 177/18410 | 0.0001 | 0.0049 |
| MF | GO，0016209 | antioxidant activity | 3/28 | 85/18410 | 0.0003 | 0.0069 |
| KEGG | hsa04218 | Cellular senescence | 4/23 | 156/8164 | 0.0009 | 0.0652 |
| KEGG | hsa04115 | p53 signaling pathway | 3/23 | 73/8164 | 0.0011 | 0.0652 |

GO，Gene Ontology; BP, biological process; CC, cellular component; MF, molecular function; KEGG, Kyoto Encyclopedia of Genes and Genomes; DEGs，Differentially expressed genes.

**Table2. GSEA analysis of TCGA-LIHC**

| ID | SetSize | EnrichmentScore | NES | Pvalue | P.adj | Qvalue |
| --- | --- | --- | --- | --- | --- | --- |
| KEGG_RIBOSOME | 85 | 0.7611006 | 3.085902 | 1e-10 | 4.38e-09 | 3.03e-09 |
| WP_CYTOPLASMIC_RIBOSOMAL_PROTEINS | 86 | 0.7587952 | 3.078273 | 1e-10 | 4.38e-09 | 3.03e-09 |
| REACTOME_INFLUENZA_INFECTION | 151 | 0.6979987 | 3.075782 | 1e-10 | 4.38e-09 | 3.03e-09 |
| REACTOME_EUKARYOTIC_TRANSLATION_ELONGATION | 89 | 0.7507659 | 3.034779 | 1e-10 | 4.38e-09 | 3.03e-09 |
| REACTOME_SRP_DEPENDENT_COTRANSLATIONAL_PROTEIN_TARGETING_TO_MEMBRANE | 109 | 0.7127232 | 2.974571 | 1e-10 | 4.38e-09 | 3.03e-09 |
| REACTOME_EUKARYOTIC_TRANSLATION_INITIATION | 116 | 0.6987621 | 2.945497 | 1e-10 | 4.38e-09 | 3.03e-09 |
| REACTOME_NONSENSE_MEDIATED_DECAY_NMD | 112 | 0.6940664 | 2.910544 | 1e-10 | 4.38e-09 | 3.03e-09 |
| REACTOME_RESPONSE_OF_EIF2AK4_GCN2_TO_AMINO_ACID_DEFICIENCY | 98 | 0.6981658 | 2.869411 | 1e-10 | 4.38e-09 | 3.03e-09 |
| REACTOME_REGULATION_OF_EXPRESSION_OF_SLITS_AND_ROBOS | 155 | 0.6393451 | 2.824605 | 1e-10 | 4.38e-09 | 3.03e-09 |
| REACTOME_CELL_CYCLE_MITOTIC | 478 | 0.5605160 | 2.806142 | 1e-10 | 4.38e-09 | 3.03e-09 |

GSEA, Gene Set Enrichment Analysis.

**Table3.** **Cox regression of dataset TCGA-LIHC**

| Characteristics | Total（N） | Univariate analysis | |  | Multivariate analysis | |
| --- | --- | --- | --- | --- | --- | --- |
|  |  | Hazard ratio （95% CI） | P value |  | Hazard ratio （95% CI） | P value |
| E2F1 | 373 | 1.231 （1.079 - 1.404） | 0.002 |  | 0.975 （0.781 - 1.218） | 0.827 |
| SERPINE1 | 373 | 1.122 （1.020 - 1.235） | 0.018 |  | 1.090 （0.966 - 1.231） | 0.163 |
| GYS2 | 373 | 0.558 （0.377 - 0.826） | 0.004 |  | 0.877 （0.533 - 1.444） | 0.606 |
| SPP1 | 373 | 1.139 （1.079 - 1.202） | < 0.001 |  | 1.057 （0.986 - 1.133） | 0.119 |
| PCK1 | 373 | 0.917 （0.854 - 0.984） | 0.016 |  | 1.081 （0.972 - 1.202） | 0.153 |
| CCNB1 | 373 | 1.457 （1.250 - 1.699） | < 0.001 |  | 1.282 （0.989 - 1.662） | 0.061 |
| CYP2C9 | 373 | 0.852 （0.793 - 0.915） | < 0.001 |  | 0.878 （0.794 - 0.971） | **0.011** |
| IGFBP3 | 373 | 1.169 （1.034 - 1.322） | 0.013 |  | 0.986 （0.848 - 1.145） | 0.848 |
| KDM8 | 373 | 0.826 （0.700 - 0.974） | 0.023 |  | 0.981 （0.794 - 1.211） | 0.857 |
| RCAN1 | 373 | 0.795 （0.642 - 0.984） | 0.035 |  | 1.129 （0.865 - 1.475） | 0.371 |
| ALPL | 373 | 0.887 （0.789 - 0.997） | 0.044 |  | 0.909 （0.790 - 1.045） | 0.181 |
| FBP1 | 373 | 0.881 （0.802 - 0.967） | 0.008 |  | 1.003 （0.879 - 1.145） | 0.963 |
| NQO1 | 373 | 1.108 （1.041 - 1.179） | 0.001 |  | 1.060 （0.983 - 1.143） | 0.128 |
| LCAT | 373 | 0.765 （0.674 - 0.867） | < 0.001 |  | 0.919 （0.785 - 1.077） | 0.296 |

TCGA, The Cancer Genome Atlas; LIHC, Liver Hepatocellular Carcinoma.

**Table4. GSVA analysis of TCGA-LIHC dataset**

| ontology | logFC | AveExpr | t | P.Value | adj.P |
| --- | --- | --- | --- | --- | --- |
| HALLMARK_ANGIOGENESIS | -0.907354821 | -0.010311404 | -12.99538088 | 9.37E-33 | 7.97E-32 |
| HALLMARK_IL2_STAT5_SIGNALING | -0.907354821 | -0.010311404 | -12.99538088 | 9.37E-33 | 7.97E-32 |
| HALLMARK_EPITHELIAL_MESENCHYMAL_TRANSITION | -0.751514215 | 0.038111566 | -12.74604327 | 9.58E-32 | 5.43E-31 |
| HALLMARK_KRAS_SIGNALING_UP | -0.756255157 | 0.048359743 | -11.90858684 | 2.02E-28 | 8.60E-28 |
| HALLMARK_MYOGENESIS | -0.595822385 | 0.072268378 | -9.040133663 | 5.56E-18 | 1.89E-17 |
| HALLMARK_G2M_CHECKPOINT | -0.601469372 | 0.01938544 | -7.833225589 | 3.83E-14 | 9.30E-14 |
| HALLMARK_PI3K_AKT_MTOR_SIGNALING | -0.601469372 | 0.01938544 | -7.833225589 | 3.83E-14 | 9.30E-14 |
| HALLMARK_REACTIVE_OXYGEN_SPECIES_PATHWAY | -0.585543805 | -0.027634564 | -7.596308483 | 1.95E-13 | 4.15E-13 |
| HALLMARK_TGF_BETA_SIGNALING | -0.415745545 | -0.002474737 | -5.284238664 | 2.02E-07 | 2.64E-07 |
| HALLMARK_COMPLEMENT | -0.415745545 | -0.002474737 | -5.284238664 | 2.02E-07 | 2.64E-07 |
| HALLMARK_INFLAMMATORY_RESPONSE | -0.415745545 | -0.002474737 | -5.284238664 | 2.02E-07 | 2.64E-07 |
| HALLMARK_UV_RESPONSE_DN | -0.415745545 | -0.002474737 | -5.284238664 | 2.02E-07 | 2.64E-07 |
| HALLMARK_COAGULATION | -0.415745545 | -0.002474737 | -5.284238664 | 2.02E-07 | 2.64E-07 |

GSVA,Gene Set Variation Analysis; TCGAthe cancer genome atlas; LIHC, Liver Hepatocellular Carcinoma.

**Table 5. List of key genes of differential expression analysis**

| Gene Symbol | Description | logFC | P.Value | adj.P.Val |
| --- | --- | --- | --- | --- |
| LCAT | lecithin-cholesterol acyltransferase | -2.83466 | 3.36E-35 | 6.10E-33 |
| CCNB1 | cyclin B1 | 2.024582 | 2.36E-32 | 3.26E-30 |
| SERPINE1 | serpin family E member 1 | -2.05142 | 7.84E-15 | 7.11E-14 |
| CYP2C9 | cytochrome P450 family 2 subfamily C member 9 | -2.45995 | 1.48E-13 | 1.11E-12 |
| NQO1 | NAD（P）H quinone dehydrogenase 1 | 2.293504 | 3.02E-09 | 1.22E-08 |
| SPP1 | secreted phosphoprotein 1 | 2.092279 | 9.64E-06 | 2.36E-05 |
